# Bile Acid Binding Resin Improves Metabolic Control through the Induction of Energy Expenditure

**DOI:** 10.1371/journal.pone.0038286

**Published:** 2012-08-29

**Authors:** Mitsuhiro Watanabe, Kohkichi Morimoto, Sander M. Houten, Nao Kaneko-Iwasaki, Taichi Sugizaki, Yasushi Horai, Chikage Mataki, Hiroyuki Sato, Karin Murahashi, Eri Arita, Kristina Schoonjans, Tatsuya Suzuki, Hiroshi Itoh, Johan Auwerx

**Affiliations:** 1 Department of Internal Medicine, School of Medicine, Keio University, Tokyo, Japan; 2 Laboratory Genetic Metabolic Diseases, Academic Medical Center, Amsterdam, The Netherlands; 3 Ecole Polytechnique Fédérale de Lausanne, Lausanne, Switzerland; 4 Department of Bioscience, Ehime University Graduate School of Medicine, Ehime, Japan; 5 Nippon Medical School, Tokyo, Japan; 6 Graduate School of Media and Governance, Keio University, Fujisawa-shi, Kanagawa, Japan; University of Tor Vergata, Italy

## Abstract

**Background:**

Besides well-established roles of bile acids (BA) in dietary lipid absorption and cholesterol homeostasis, it has recently become clear that BA is also a biological signaling molecule. We have shown that strategies aimed at activating TGR5 by increasing the BA pool size with BA administration may constitute a significant therapeutic advance to combat the metabolic syndrome and suggest that such strategies are worth testing in a clinical setting. Bile acid binding resin (BABR) is known not only to reduce serum cholesterol levels but also to improve glucose tolerance and insulin resistance in animal models and humans. However, the mechanisms by which BABR affects glucose homeostasis have not been established. We investigated how BABR affects glycemic control in diet-induced obesity models.

**Methods and Findings:**

We evaluated the metabolic effect of BABR by administrating colestimide to animal models for the metabolic syndrome. Administration of BABR increased energy expenditure, translating into significant weight reduction and insulin sensitization. The metabolic effects of BABR coincide with activation of cholesterol and BA synthesis in liver and thermogenesis in brown adipose tissue. Interestingly, these effects of BABR occur despite normal food intake and triglyceride absorption. Administration of BABR and BA had similar effects on BA composition and thermogenesis, suggesting that they both are mediated *via* TGR5 activation.

**Conclusion:**

Our data hence suggest that BABR could be useful for the management of the impaired glucose tolerance of the metabolic syndrome, since they not only lower cholesterol levels, but also reduce obesity and improve insulin resistance.

## Introduction

Bile acid (BA) is essential constituents of bile that facilitate dietary lipid absorption and cholesterol catabolism. BA also activates several signaling pathways, endowing them with an endocrine function. For instance, BA was shown to be natural ligands that activate the nuclear receptor farnesoid X receptor (FXR, NR1H4) [1]–[3], which controls both the synthesis and enterohepatic circulation of BA [4]–[5]. FXR induces the expression of the short heterodimer partner (SHP, NR0B2), an atypical nuclear receptor that acts as a corepressor. The FXR-mediated SHP induction contributes to the negative feedback regulation of BA biosynthesis, through inhibition of liver X receptor α and β (LXRα, NR1H3 and LXRβ, NR1H2) and liver receptor homolog-1 (LRH-1, NR5A2), both required for the transcription of the rate-limiting enzyme in the neutral BA biosynthesis pathway, cholesterol 7α-hydroxylase (CYP7A1) [6]–[11]. The FXR-mediated induction of FGF15/19 (FGF19 in human and its ortholog FGF15 in mouse) in intestinal epithelial cells also participates in the feedback repression of BA synthesis, via FGFR4 on hepatocytes [12]–[14]. Using a similar mechanism, the FXR-mediated SHP induction attenuates the capacity of LXR and LRH-1 to induce the expression of sterol regulatory element-binding protein (SREBP)-1c, the master regulator of lipogenesis, explaining the inhibition of hepatic fatty acid and triglyceride biosynthesis and VLDL production by BA administration [15]. Recently, it was reported that FXR deficiency improves glucose homeostasis in a mouse model for the metabolic syndrome [16]. In addition, we established that a synthetic FXR agonist (GW4064), deteriorates metabolic control in a diet-induced obesity mouse model [17]. These results suggest that the BA-specific nuclear receptor FXR is involved in the pathogenesis of the metabolic syndrome.

BA may also signal in peripheral tissues through another pathway involving the binding and activation of TGR5, a G protein-coupled receptor (GPCR), leading to the induction of intracellular cyclic adenosine monophosphate (cAMP) levels [18]–[19]. The subsequent activation of type 2 iodothyronine deiodinase (D2), the enzyme which converts inactive thyroxine into active 3,5,3′-triiodothyronine [20] and hence determines thyroid hormone receptor saturation in cells, and of peroxisome proliferator-activated receptor (PPAR) γ coactivator-1α (PGC-1α), the master regulator of mitochondrial biogenesis [21], then stimulates energy expenditure in brown adipose tissue (BAT) (in rodents) and skeletal muscle (in humans) [22].Activation of this pathway explains how administration of BA to mouse models of obesity and diabetes induces weight loss and insulin sensitization. In addition, we reported that in mice, a synthetic FXR agonist (GW4064) reduced the BA pool and altered BA composition impairing peripheral energy metabolism possibly *via* TGR5 [17]. Furthermore, TGR5 activation enhances GLP-1 secretion from the enteroendocrine L-cell stimulating pancreatic insulin secretion [23]. Thus in addition to FXR, the BA-specific GPCR TGR5, is an attractive therapeutic target for treating metabolic syndrome.

These observations have built a strong case that BA has effects beyond the strict control of BA homeostasis and function as general metabolic integrators [24]. Bile acid binding resins (BABR), such as cholestyramine, is effective drugs for the treatment of coronary heart disease by lowering LDL-cholesterol as primary prevention, and for the treatment of cholestatic liver disease. BABR absorbs BA in the intestine thereby preventing their uptake in the ileum and interrupting their enterohepatic circulation. The resulting decrease of negative feedback signals will induce the expression of *Cyp7a1*. The subsequent decrease in intrahepatic cholesterol levels will on its turn activate SREBP-2, which induces the expression of the low density lipoprotein (LDL) receptor, to enhance cholesterol uptake, and of enzymes that synthesize cholesterol de novo, such as 3-hydroxy-3-methylglutaryl (HMG) CoA reductase. BABR was also reported to improve glycemic control in a type 2 diabetes mouse model [25], but the mechanism has not been established. We characterize here in detail the molecular and functional impact of a second generation BABR, colestimide [26], on metabolic homeostasis in animal models for the metabolic syndrome. Interestingly, colestimide not only reduces cholesterol levels but also decreases body weight and improves glucose tolerance, qualifying BABR as ideal agents to treat the metabolic syndrome. We suggest that a part of the anti-metabolic syndrome effect of BABR will be exerted by an alteration of the peripheral BA composition followed by TGR5 activation.

## Materials and Methods

### Materials

Cholic acid (CA) and cholestyramine were obtained from Sigma (St. Quentin Fallavier, France). Colestimide was a generous gift of Mitsubishi Pharmaceuticals.

### Animal studies

All procedures undertaken in the present study conformed to the principles outlined in the *Guide for the Care and Use of Laboratory Animals* published by the USA National Institutes of Health (NIH Publication No. 85-23, revised 1996) and were approved by the Institutional Animal Care and Use Committee of Keio University School of Medicine (permission No. 08062-(2)). Male C57BL/6J mice, 6–7 weeks of age, were obtained from Charles River Laboratories France (l'Arbresle, France) and CLEA Japan Inc. (Tokyo, Japan), respectively. All mice were maintained in a temperature-controlled (23°C) facility with a 12 hours light/dark cycle and were given free access to food and water. The high-fat diet was obtained from Research diets (New Jersey, USA). The high-fat diet (D12492) contained 20 kcal% protein, 20 kcal% carbohydrate and 60 kcal% fat. For treatment with BA or BABR, mice were fed diets mixed with CA (0.5% w/w) or colestimide (2% w/w). Based on a daily food intake of 5 g, this resulted in a daily dose of colestimide 100 mg. The mice were fasted 4 hours before harvesting blood for subsequent blood measurements, and tissues for RNA isolation, lipid measurements and histology. Food intake was measured from the accumulated weight of the food for 1 week, with 5 mice in each group. Oxygen consumption was measured using the Oxymax apparatus (Columbus Instruments, Columbus, OH) [27].

### Morphological studies

Pieces of mouse tissues were fixed in Bouin's solution, dehydrated in ethanol, embedded in paraffin, and cut at a thickness of 5 µm. Sections were deparaffinized, rehydrated, and stained with haematoxylin and eosin.

### mRNA expression analysis by Q-RT-PCR

Expression levels were analyzed in cDNA synthesized from total mRNA using real-time PCR as described [22]. The sequences of the primer sets used are displayed in table 1.

**Table 1:** Primer sequences of genes used for quantification of mRNAs by real-time PCR.

Gene	Forward Primer (5′→3′)	Reverse Primer (5′→3′)
*18s*	GATGGGAAGTACAGCCAGGT	TTTCTTCAGCCTCTCCAGGT
*Cyp7A1*	TACAGAGTGCTGGCCAAGAG	TTCAAGGATGCACTGGAGAG
*SHP*	CAAGGAGTATGCGTACCTGAAG	GGCTCCAAGACTTCACACAGT
*FXR*	CAAAATGACTCAGGAGGAGTACG	GCCTCTCTGTCCTTGATGTATTG
*PGC-1a*	AAGGGCCAAACAGAGAGAGA	GCGTTGTGTCAGGTCTGATT
*PEPCK*	GGGAACTCACTACTCGGGAA	GCCAGGTATTTCTTCTTGCC
*G6Pase*	CCGGATCTACCTTGCTGCTCACTTT	TAGCAGGTAGAATCCAAGCGCGAAAC
*SREBP-2*	AAGTGACCGAGAGTCCCTTG	ACGTTGAGACTGCTCCACAG
*HMGCR*	TCGAAGGACGAGGAAAGACT	CGTCAACCATAGCTTCCGTA
*LDLR*	AGGCTGTGGGCTCCATAGG	TGCGGTCCAGGGTCATCT
*PPARa*	GGTGAGGAGAGCTCTGGAAG	GAAGCTGGAGAGAGGGTGTC
*ACC*	ACCCACTCCACTGTTTGTGA	CCTTGGAATTCAGGAGAGGA
*SCD1*	CTCCTGCTGATGTGCTTCAT	AAGGTGCTAACGAACAGGCT
*D2*	TTCTGAGCCGCTCCAAGT	GGAGCATCTTCACCCAGTTT
*UCP-1*	GGCCCTTGTAAACAACAAAATAC	GGCAACAAGAGCTGACAGTAAAT
*FGF15*	GGCAAGATATACGGGCTGAT	GATGGTGCTTCATGGATCTG
*mCPT-1*	GCACTGCAGCTCGCACATTACAA	CTCAGACAGTACCTCCTTCAGGAAA
*Cyp8B1*	GGAAGCCAAGAAGTCGTTCA	GACGCAGACTCTCCTCCATC
*Cyp27A1*	TCTGGCTACCTGCACTTCCT	CTGGATCTCTGGGCTCTTTG

### Clinical biochemistry and evaluation of glucose and lipid homeostasis

An oral glucose tolerance test (OGTT) was performed in animals that were fasted overnight. Glucose was administered by gavage at a dose of 2 g/kg. An intra peritoneal insulin tolerance test (IPITT) was done in 4 h fasted animals. Insulin was injected at a dose of 0.75 U/kg. Glucose quantification was done with the Maxi Kit Glucometer 4 (Bayer Diagnostic, Puteaux, France) or Glucose RTU (bioMérieux Inc., Marcy l'Etoile, France). Plasma insulin concentrations were measured using ELISA for mouse (Cristal Chem Inc., Downers Grove, IL). HOMA-R was calculated by this formula: (fasting serum insulin concentration [µU/ml])*(fasting serum glucose concentration [mg/dl])/405. Free fatty acids, triglycerides, and total cholesterol were determined by enzymatic assays (Roche, Mannheim, Germany). LDL cholesterol was measured with plasma clinical chemistry analysis using AU-400 automated laboratory workstation and commercial reagents (Olympus France SA, Rungis, France) [28]. BA in enterohepatic organs were determined as described [29]. Lipid absorption was calculated as follows; (food lipid content – fecal lipid content)/food lipid content (×100(%)), with the food and feces consumed by/accumulated from 5 mice for 48 hours. To calculate the lipid absorption, TG was extracted from the accumulated feces by the classical Folch method [30] and measured as previously described [15].

### Statistical analysis

Values were reported as mean +/− standard error (SEM). Statistical differences were determined using ANOVA (Statsview software, Abacus concepts, Inc., Berkeley, CA). Statistical significance is displayed as * (*P*<0.05) or ** (*P*<0.01) versus F.

## Results

### BABR prevents the onset of diet-induced obesity

**Figure 1:**
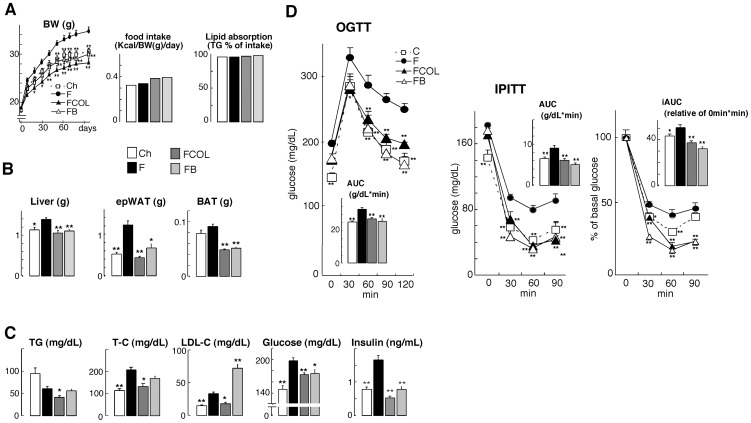
BA and BABR improve metabolic control in DIO C57BL/6J mice model. (A) Body weight, food intake and TG absorption (B) Liver, epididymal WAT (epWAT), and BAT weight change of C57BL/6J mice during 96 days on different diets. Ch stands for chow, F denotes HF diet, FCOL denotes HF diet+2.0% w/w colestimide and FB denotes HF diet+0.5% w/w CA. (C) Serum levels of TG, T-C, LDL-C, glucose and insulin in C57BL/6J mice on the indicated treatments. (D) Glucose levels during OGTT and IPITT in the different treatment groups (AUC is depicted in the inset). The OGTT were performed after an overnight fast after 9 weeks of administration. Glucose was administered by gavage at a dose of 2 g/kg. The IPITT were performed after 4 h fast after 10 weeks of administration. Insulin was injected at a dose of 0.75 U/kg. Data are expressed as the mean +/− SEM (n = 5–6). * (*P*<0.05) or ** (*P*<0.01) versus F.

To evaluate the metabolic effects of a BABR in models of diet-induced obesity (DIO), we fed C57BL/6J mice normal chow, HF diet, or HF diet supplemented with either colestimide (2% w/w) or cholic acid (CA, 0.5% w/w) for 96 days. HF fed animals gained more weight than chow-fed animals. The animals fed with CA supplemented HF diet gained weight at a rate comparable to chow-fed mice. Colestimide had an even more pronounced effect in curbing weight gain. Since food intake and lipid absorption were not affected by CA and colestimide, these effects on body weight are probably mediated by increased energy expenditure (Fig. 1A). At necropsy, the weight of liver, epididymal white adipose tissue (epWAT) and BAT of HF fed animals was all significantly increased (Fig. 1B). The BAT was paler, indicative of increased fat accumulation, and there was an expansion of WAT surrounding the BAT (not shown). Both colestimide and CA completely prevented HF-induced changes in liver and adipose mass and morphology. High fat diet-induced significantly increased serum total cholesterol (T-C), LDL-cholesterol (LDL-C), fasting glucose and insulin levels. Colestimide ameliorated serum triglyceride (TG), T-C, LDL-C, fasting glucose and insulin levels significantly. CA administration exerted significant reduction of fasting glucose and insulin levels, but induced serum LDL-C level as expected (Fig. 1C). During OGTT, both colestimide and CA significantly reduced blood glucose concentrations to normalize the glucose tolerance of the mice with diet-induced obesity. Insulin sensitivity of the mice was also improved by colestimide and CA, shown in the result of IPITT (Fig. 1D). In KK-***A^y^*** mice, both colestimide and cholestiramine improved metabolic status (Text S1) without suppressing their food intake (Fig. S1A). The BABR significantly reduced epWAT weight gain, and also significantly improved serum metabolic index including TG, FFA, fasting glucose, insulin levels, and HOMA-R. Colestimide administration significantly improved liver weight gain and serum T-C level, either (Fig. S1B–D). The BABR-received KK-***A^y^*** mice exhibited significantly lower blood glucose during the OGTT (Fig. S1E). In the IPITT, the BABR reduced blood glucose level, but the improvement rate described in iAUC was not affected by the BABR administration (Fig. S1E). These data show that BABR improves metabolic control in mouse models for the metabolic syndrome.

### BABR increases energy expenditure

**Figure 2:**
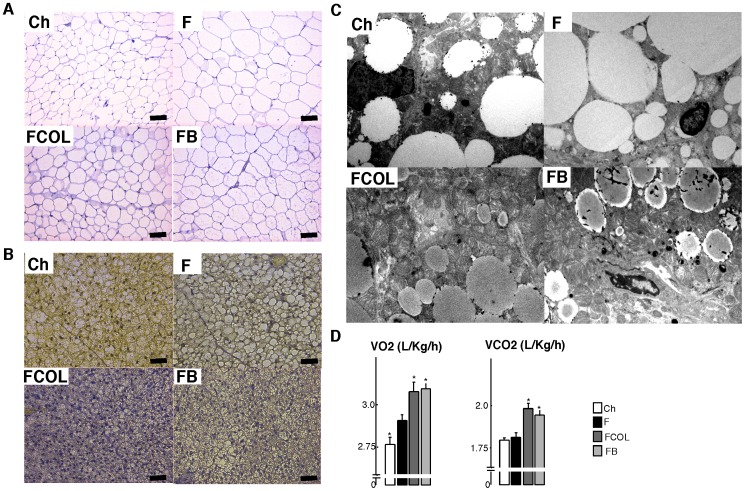
BABR increase energy expenditure. Hematoxylin and eosin (HE) stained epWAT (A) and BAT (B) sections of C57BL/6J animals treated with control or HF diet when indicated combined with colestimide or CA as specified in Fig. 1. Scale bar, 50 µm. (C) BAT analysis by transmission electron microscopy. (D) Averaged O_2_ consumption (VO_2_) and CO_2_ production (VCO_2_) as measured by indirect calorimetry in mice on the different diets as indicated. Data are expressed as the mean +/− SEM (n = 5–6). * (*P*<0.05) or ** (*P*<0.01) versus F.

The significant weight loss, in the wake of an unaltered food intake, suggested that BABR could stimulate energy expenditure and as such improve metabolic homeostasis. We hence analyzed the morphology of key metabolic tissues and performed indirect calorimetry in the C57BL/6J mice used in the HF study (see Fig. 1). This furthermore enabled us to compare the effect of the BABR with those of BA, which we characterized previously in this model [15] [22]. Liver sections of HF fed animals showed more unstained inclusions, indicative of steatosis, which were absent when the HF diet was supplemented with colestimide or CA (not shown and [15]). The HF diet induced significant adipocyte hypertrophy in both epWAT, characterized by a larger adipocyte volume (Fig. 2A), and BAT, typified by larger lipid vacuoles within the cells. This adipocyte hypertrophy was not observed when the HF diet was supplemented with either colestimide or CA. Electron microscopic analysis of BAT also showed more lipid vacuoles in HF fed animals when compared with chow fed animals or animals receiving HF diet combined with colestimide or CA (Fig. 2A,B&C). Compared with the HF diet, colestimide and CA supplementation increased the number of lamellar cristae in the mitochondria (Fig. 2C). Indirect calorimetry, showed a higher CO_2_ production and O_2_ consumption in animals fed a HF diet with either colestimide or CA when compared to animals on a HF or a normal diet (Fig. 2D). We conclude from these experiments that feeding of colestimide or CA changes fat and energy metabolism most likely due to an effect on basal metabolic rate.

### Molecular mechanism of BABR action

**Figure 3:**
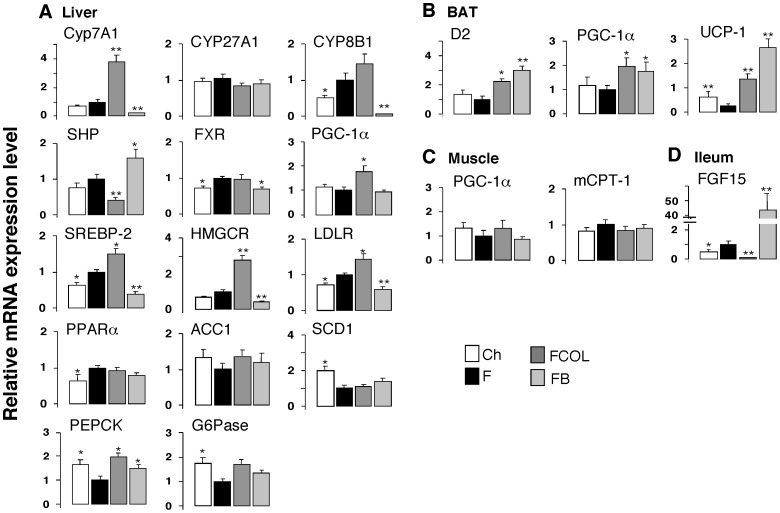
Gene expression in liver, BAT, muscle and ileum. (A) mRNA expression levels of *Cyp7a1*, *Cyp8b1, Cyp27a1, Shp*, *Fxr*, *Pgc-1α*, *Pepck*, *G6Pase*, *Srebp-2*, HMG-CoA reductase, LDL-Receptor, *Pparα*, *Acc1* and *Scd1* were determined using quantitative RT-PCR in liver of C57BL/6J mice treated as described in Fig. 1A. (B) mRNA expression levels of *D2*, *Pgc-1α* and *Ucp-1* in BAT. (C) *Pgc-1α* and *mCpt-1* in muscle. (D) *Fgf15* in ileum. Treatments and abbreviations are identical to those specified in Fig. 1A. Mice were fasted 4 hours before sacrifice and tissue collection. Data are expressed as the mean +/− SEM (n = 5–6). * (*P*<0.05) or ** (*P*<0.01) versus F.

To identify the molecular drivers of the effects of BABR, we performed analysis of gene expression using Q-RT-PCR in liver, BAT, muscle and ileum of the C57BL/6J HF study (Fig. 1). Hepatic gene expression reflected the interruption of the enterohepatic cycle of BA by BABR and its consequences on BA production and cholesterol homeostasis. These changes were typified by the significant induction of *Cyp7a1* expression, subusequent to the reduction in *Shp*. *Cyp8b1* expression was significantly suppressed by CA administration, but was not affected by BABR administration. Gene expression of *Cyp27a1*, another rate-limiting enzyme participating in the alternative acidic BA synthesis pathway, was not affected by CA or BABR administration. The cholesterol depletion caused by colestimide stimulated BA production by CYP7A1, and then induced significantly increased expression levels of *Srebp-2* and its target genes including HMG-CoA reductase and LDL receptor. Genes involved in gluconeogenesis, such as phosphoenolpyruvate carboxykinase (*Pepck*) and glucose-6-phophatase (*G6Pase*) were affected. *Pepck* gene expression was significantly induced, and *G6Pase* gene expression was also stimulated as a consequence of the significant rise in the *Pgc-1a* expression, which stimulates gluconeogenesis. In contrast to colestimide, and as previously reported, the FXR agonist CA significantly induced *Shp* mRNA levels, which attenuates the expression of *Cyp7a1* and of the genes involved in cholesterol homeostasis. Genes involved in fatty acid oxidation (*Pparα*) and lipogenesis (acetyl-CoA carboxylase 1 (*Acc1*) and stearoyl-CoA desaturase-1 (*Scd1*)) were not changed in response to colestimide and CA (Fig. 3A).

**Figure 4:**
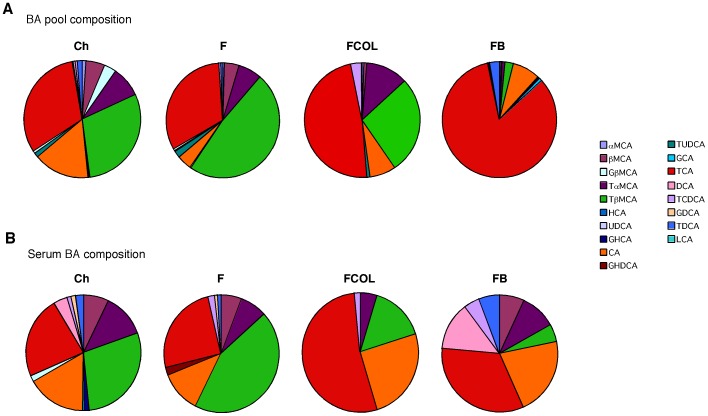
Bile acid composition in the enterohepatic organs and serum. Bile acid composition in the enterohepatic organs and serum of C57BL/6J fed with high fat diet (Fig. 1A) after treatment with colestimide or CA. Undefined abbreviations are: G, glycol; T, tauro; CD, chenodeoxy; D, deoxy; H, hyo; HD, hyodeoxy; UD, ursodeoxy; L, litho; M, muri.

In BAT, the expression of *Pgc-1α* and *D2* were both induced by colestimide and CA. As a consequence the expression of uncoupling protein-1 (*Ucp-1*) was also increased after both BABR and BA administration (Fig. 3B). Colestimide and CA treatment did not lead to significant differences in the expression of the genes involved in energy homeostasis in muscle (Fig. 3C). In ileum, the expression of *Fgf15*, which is one of the target genes of FXR, was significantly decreased by colestimide and increased by CA (Fig. 3D). In combination, the gene expression studies confirm that BAT and liver are the primary target organs that contribute to the beneficial effects of BABR on energy, lipid and glucose homeostasis. Remarkably, the effect on energy homeostasis induced by BABR was very similar to those observed after administration of CA (Figs. 1, 2, 3, Fig. S1 and [22]), despite the fact that BABR and BA has opposite actions on hepatic gene expression. Precisely, BABR administration induces *Cyp7a1* expression, while BA supplementation reduces expression of this gene, which is secondary to the changes in the BA pool size and serum BA levels. CA increased the BA pool and serum BA levels, but unexpectedly colestimide induced only a minor and non significant decrease in these parameters (Table 2). More striking, the changes in the composition of the BA pool and serum BA was similar after BABR or CA administration [22]. Both treatments increased the relative contribution of CA and its derivatives, most notably tauroCA. Bile acids derived from chenodeoxyCA such as tauromuriCA were decreased by both BA supplementation and BABR treatment (Fig. 4 and [22]). BABR preferentially sequesters mono- and di-hydroxy BA and prevent them from enterohepatic recirculation hence inducing the de novo synthesis of CA.

**Table 2:** BA pool size and serum BA concentration in C57BL/6J mice.

	BA pool size (nmol/g Liver+Intestine)	Serum BA (µM)
Ch	9852.6+/−191.2^A^	15.23+/−2.23
F	9130.4+/−261.6	13.83+/−1.80
FCOL	8643.4+/−254.8	11.84+/−1.68
FB	22867.0+/−145.3^B^	18.21+/−1.16^A^

Ch denotes chow, F denotes HF diet, FCOL denotes HF diet+2% w/w colestimide and FB denotes HF diet+0.5% w/w CA as specified in Fig. 1A.

Data are expressed as mean +/− SEM (n = 5–6).

A *P*<0.05 versus F.

B *P*<0.01 versus F.

## Discussion

In the present study, we show that administration of BABR stimulated energy expenditure mediated by BAT, thereby preventing and reversing diet-induced obesity in mice. This phenomenon was accompanied by an improved glucose tolerance and insulin sensitization in a diet-induced obesity model (C57BL6/J (Fig. 1)). BABR also improved glucose tolerance in KK-***A^y^*** mice (Text S1 and Fig. S1). Brown adipose tissue is recently recognized as an important tissue of thermogenesis and energy homeostasis not only in rodents but also in man [31]–[33]. Our results indicate that therapy with colestimide, a new and better formulated BABR when compared with cholestyramine, could improve metabolic control also in humans suffering from the metabolic syndrome. In fact, colestimide decreased fasting glucose levels, but also reduced body weight, BMI, and visceral fat mass [34]. Furthermore, BABR was reported to improve obesity, insulin sensitivity and glycemic control in diabetes mellitus mouse model [25]. Furthermore, there is clinical evidence suggesting that BABR such as colesevelam may improve both lipid control and glycemic control in patients with type 2 diabetes that receive oral antihyperglycemic medications [35]–[37] [38], [39]. Combined with the limited systemic toxicity ofBABR, which isnot absorbed, these compounds could constitute a significant advance in our therapeutic armamentarium to combat against metabolic syndrome. Although there is some evidence that the beneficial effects of BABR may be mediated through FXR, LXR, FGF15/19, and TGR5, but the exact molecular mechanisms are not yet clearly defined.

**Figure 5:**
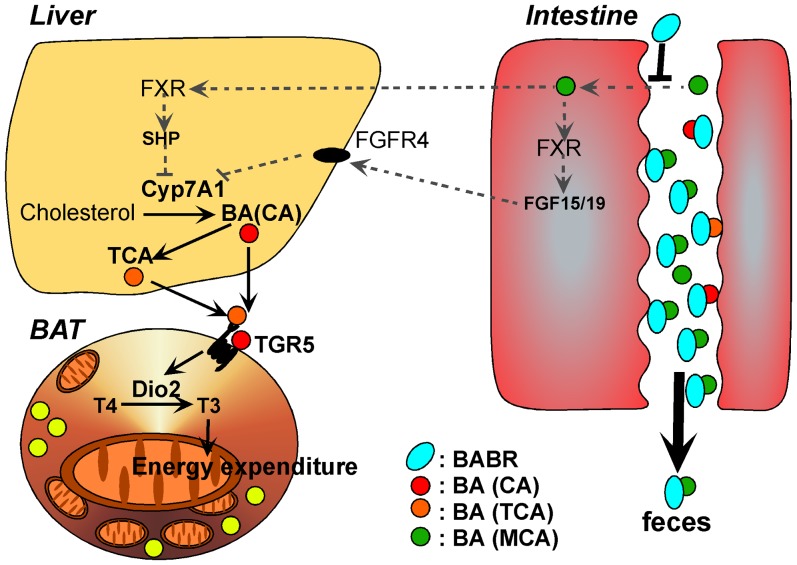
Changes in energy metabolism by BABR administration. Administration of BABR to animals leads to induction of bile acid synthesis and as a consequence a relative increase in CA and TCA. This translates into induced energy expenditure in brown adipose tissue, hence improving obesity and diabetes.

On a molecular and cellular level, BABR improves metabolic homeostasis through effects on liver and BAT (Fig. 5). The effects on liver are well known and include an induction of cholesterol and BA biosynthesis, subsequent to the fecal loss of bile acids caused by the BABR treatment. This underlies the cholesterol-lowering effect of BABR. The metabolic effects on BAT have not been reported before and phenocopy the changes seen after treatment of rodents with primary BA, such as CA [22]. This is surprising, since treatment with BABR and BA has opposite actions on most parameters of BA homeostasis. CA administration increases FXR activation, whereas BABR treatment decreases it. CA administration decreases the BA synthesis, whereas BABR treatment increases it. However, BABR and CA had similar effects on BA composition. Both treatments increased the relative contribution of CA and its derivatives, most notably deoxyCA, tauro-deoxyCA and tauroCA. Bile acids derived from chenodeoxyCA such as muriCA and tauromuriCA were decreased. In addition to our studies in mice, colestimide treatment of hypercholesterolemia patients significantly increased CA in bile [40]. The most likely explanation for this specific increase in BA species derived from CA, is the fact that colestimide has a high adsorptive capacity for mono- and di-hydroxy BA like chenodeoxyCA and lithoCA, but a relative low capacity for the tri-hydroxy BA such as CA. In addition, the induced BA biosynthesis during colestimide treatment might produce more CA than chenodeoxyCA [41]. Increased BA pool size and plasma BA levels are fine indicator for TGR5 activation [22] [17]. This time, we focus on the importance of bile acid composition to improve metabolic status. TGR5 is activated by almost all BA including mono- and di-hydroxy BA. Some of the BA like ursodeoxyCA have little activity on TGR5, but no inhibitory BA was identified in our test of over 60 BA and BA derivatives for antagonistic effects on TGR5 (data not shown). Most importantly, BABR administration induced levels of tauroCA, a relatively potent TGR5 agonist [22], which could be the key to the anti-metabolic syndrome effect of BABR administration. In agreement with this, reduction of BA pool size and tauroCA levels by the administration of the synthetic FXR agonist GW4064, exacerbated the effects of HF feeding [17].

It is conceivable that BABR, such as colestimide and cholestyramine that are mainly active in the intestinal tract, could affect the production of incretins, such as FGF15, cholecystokinin (CCK) and glucagon like peptide-1 (GLP-1). FGF15 is interesting in this respect, since transgenic mice that overexpress the human *Fgf15* ortholog *Fgf19* in the muscle or in a more general pattern have increased metabolic rate and decreased adiposity [42]. BABR, however, decreases expression of *Fgf15* (Fig. 3D), whereas CA has the opposite effect, making it unlikely that it underlies the common metabolic effects of BABR. GLP-1 has glucose-dependent insulinotropic actions on the pancreatic beta-cells and has recently been associated with bile acids because its release was stimulated in an enteroendocrine cell line via TGR5, a GPCR specific for bile acids [23]. BABR may have an effect on the intestinal secretion of GLP-1, according to recent reports [43]. Indeed, we found that GLP-1 secretion was stimulated by BABR administration (unpublished data), which may contribute to the other beneficial effects of BABR. CCK is another good candidate, since cholestyramine can increase CCK production but also pancreatic beta cell function [44]–[45]. In addition CCK has been linked to increased sympathetic activity to BAT [46]–[47]. To date, effects of BABR on incretins and BAT have not been sufficiently studied.

Taken together, our data show that BABR activates energy expenditure, resulting in weight loss and improved glucose tolerance in animal models suffering from the metabolic syndrome, in a mechanism very similar to BA administration [22]. The alteration of BA composition, which occurs after BABR and CA administration, more specifically the increase of tauroCA, may be the key to improve the metabolic status. A recent study in man employing colesevalam treatment in type 2 diabetic patients revealed no correlation energy expenditure with plasma BA levels [48]. Another report showed that BA kinetics caused by BABR administration could not affect the improvement of glycemic control in patients with T2DM [49]. In fact, biophysiological roles and significances of the each composition of the BA profiles might not be identical in human and mice, and it is difficult to make a plain comparison between the report and our result. Futhermore, in the report, the ‘BA kinetics’ was just a cholic acid or total bile acid synthesis. More precise analysis according to BA profiles, as we performed in this article, would provide a clue to solve the mechanism of improved glycemic control by BABR. These reports illustrate that the mechanisms involved in the beneficial effects of BABR in humans are still controversial and further investigation is warranted. Our previous study demonstrated one of the various mechanisms of BABR in anti-metabolic syndrome effect. Our findings in mice could be useful clues to elucidate the signaling functions of BA in man.

## Supporting Information

Figure S1 **BABR improves metabolic control in KK-** ***A^y^*** **mice.** (A) Body weight (BW) and food intake change of KK-*A^y^* mice. Ch denotes chow, COL denotes chow+colestimide and CHO denotes chow+cholestyramine. (B) A comparison of the weight of liver, epididymal WAT (epWAT) and BAT fat pads after the different interventions. (C) Serum levels of triglycerides (TG), free fatty acids (FFA), total cholesterol (T-C) in KK-*A^y^* mice on the indicated treatments. (D) Serum levels of glucose and insulin in KK-*A^y^* mice on the indicated treatments. The HOMA-IR is calculated as described in the materials and methods. (E) Glucose levels during an OGTT and IPITT, and area under the curve (AUC) and integrated areas under the curve (iAUC) in KK-*A^y^* mice in the different treatment groups. The OGTT were performed after an overnight fast after 2 weeks of administration. Glucose was administered by gavage at a dose of 1 g/kg. The IPITT were performed after 4 hours fast after 3 weeks of administration. Insulin was injected at a dose of 0.75 U/kg. Data are expressed as the mean +/− SEM (n = 5–6). ^#^ (*P*<0.05) or ^##^ (*P*<0.01) versus Ch. Further description about the materials and methods of the experiments is included in “Materials and Methods S1”.
(DOC)

Text S1 (DOCX)

Materials and Methods S1 (DOC)

## References

[1] Makishima M, Okamoto AY, Repa JJ, Tu H, Learned RM, et al (1999) Identification of a nuclear receptor for bile acids. Science 284: 1362–1365. 10334992 10.1126/science.284.5418.1362

[2] Parks DJ, Blanchard SG, Bledsoe RK, Chandra G, Consler TG, et al (1999) Bile acids: natural ligands for an orphan nuclear receptor. Science 284: 1365–1368. 10334993 10.1126/science.284.5418.1365

[3] Wang H, Chen J, Hollister K, Sowers LC, Forman BM (1999) Endogenous bile acids are ligands for the nuclear receptor FXR/BAR. Mol Cell 3: 543–553. 10360171 10.1016/s1097-2765(00)80348-2

[4] Houten SM, Auwerx J (2004) The enterohepatic nuclear receptors are major regulators of the enterohepatic circulation of bile salts. Ann Med 36: 482–491. 15513299 10.1080/07853890410018790

[5] Russell DW (2003) The enzymes, regulation, and genetics of bile acid synthesis. Annu Rev Biochem 72: 137–174. 12543708 10.1146/annurev.biochem.72.121801.161712

[6] Brendel C, Schoonjans K, Botrugno OA, Treuter E, Auwerx J (2002) The small heterodimer partner interacts with the liver X receptor alpha and represses its transcriptional activity. Mol Endocrinol 16: 2065–2076. 12198243 10.1210/me.2001-0194

[7] Goodwin B, Jones SA, Price RR, Watson MA, McKee DD, et al (2000) A regulatory cascade of the nuclear receptors FXR, SHP-1, and LRH-1 represses bile acid biosynthesis. Mol Cell 6: 517–526. 11030332 10.1016/s1097-2765(00)00051-4

[8] Kerr TA, Saeki S, Schneider M, Schaefer K, Berdy S, et al (2002) Loss of nuclear receptor SHP impairs but does not eliminate negative feedback regulation of bile acid synthesis. Dev Cell 2: 713–720. 12062084 10.1016/s1534-5807(02)00154-5 PMC4010195

[9] Lu TT, Makishima M, Repa JJ, Schoonjans K, Kerr TA, et al (2000) Molecular basis for feedback regulation of bile acid synthesis by nuclear receptors. Mol Cell 6: 507–515. 11030331 10.1016/s1097-2765(00)00050-2

[10] Sinal CJ, Tohkin M, Miyata M, Ward JM, Lambert G, et al (2000) Targeted disruption of the nuclear receptor FXR/BAR impairs bile acid and lipid homeostasis. Cell 102: 731–744. 11030617 10.1016/s0092-8674(00)00062-3

[11] Wang L, Lee YK, Bundman D, Han Y, Thevananther S, et al (2002) Redundant pathways for negative feedback regulation of bile acid production. Dev Cell 2: 721–731. 12062085 10.1016/s1534-5807(02)00187-9

[12] Holt JA, Luo G, Billin AN, Bisi J, McNeill YY, et al (2003) Definition of a novel growth factor-dependent signal cascade for the suppression of bile acid biosynthesis. Genes Dev 17: 1581–1591. 12815072 10.1101/gad.1083503 PMC196131

[13] Inagaki T, Choi M, Moschetta A, Peng L, Cummins CL, et al (2005) Fibroblast growth factor 15 functions as an enterohepatic signal to regulate bile acid homeostasis. Cell Metab 2: 217–225. 16213224 10.1016/j.cmet.2005.09.001

[14] Stroeve JH, Brufau G, Stellaard F, Gonzalez FJ, Staels B, et al (2010) Intestinal FXR-mediated FGF15 production contributes to diurnal control of hepatic bile acid synthesis in mice. Lab Invest 90: 1457–1467. 20531290 10.1038/labinvest.2010.107 PMC6643294

[15] Watanabe M, Houten SM, Wang L, Moschetta A, Mangelsdorf DJ, et al (2004) Bile acids lower triglyceride levels via a pathway involving FXR, SHP, and SREBP-1c. J Clin Invest 113: 1408–1418. 15146238 10.1172/JCI21025 PMC406532

[16] Prawitt J, Abdelkarim M, Stroeve JH, Popescu I, Duez H, et al (2011) Farnesoid x receptor deficiency improves glucose homeostasis in mouse models of obesity. Diabetes 60: 1861–1871. 21593203 10.2337/db11-0030 PMC3121443

[17] Watanabe M, Horai Y, Houten SM, Morimoto K, Sugizaki T, et al (2011) Lowering bile acid pool size with a synthetic FXR agonist induces obesity and diabetes through reduced energy expenditure. J Biol Chem 10.1074/jbc.M111.248203 PMC3143650 21632533

[18] Kawamata Y, Fujii R, Hosoya M, Harada M, Yoshida H, et al (2003) A G protein-coupled receptor responsive to bile acids. J Biol Chem 278: 9435–9440. 12524422 10.1074/jbc.M209706200

[19] Maruyama T, Miyamoto Y, Nakamura T, Tamai Y, Okada H, et al (2002) Identification of membrane-type receptor for bile acids (M-BAR). Biochem Biophys Res Commun 298: 714–719. 12419312 10.1016/s0006-291x(02)02550-0

[20] Bianco AC, Salvatore D, Gereben B, Berry MJ, Larsen PR (2002) Biochemistry, cellular and molecular biology, and physiological roles of the iodothyronine selenodeiodinases. Endocr Rev 23: 38–89. 11844744 10.1210/edrv.23.1.0455

[21] Puigserver P, Spiegelman BM (2003) Peroxisome proliferator-activated receptor-gamma coactivator 1 alpha (PGC-1 alpha): transcriptional coactivator and metabolic regulator. Endocr Rev 24: 78–90. 12588810 10.1210/er.2002-0012

[22] Watanabe M, Houten SM, Mataki C, Christoffolete MA, Kim BW, et al (2006) Bile acids induce energy expenditure by promoting intracellular thyroid hormone activation. Nature 439: 484–489. 16400329 10.1038/nature04330

[23] Thomas C, Gioiello A, Noriega L, Strehle A, Oury J, et al (2009) TGR5-mediated bile acid sensing controls glucose homeostasis. Cell Metab 10: 167–177. 19723493 10.1016/j.cmet.2009.08.001 PMC2739652

[24] Houten SM, Watanabe M, Auwerx J (2006) Endocrine functions of bile acids. Embo J 25: 1419–1425. 16541101 10.1038/sj.emboj.7601049 PMC1440314

[25] Kobayashi M, Ikegami H, Fujisawa T, Nojima K, Kawabata Y, et al (2007) Prevention and treatment of obesity, insulin resistance, and diabetes by bile acid-binding resin. Diabetes 56: 239–247. 17192488 10.2337/db06-0353

[26] Homma Y, Kobayashi T, Yamaguchi H, Ozawa H, Sakane H, et al (1997) Specific reduction of plasma large, light low-density lipoprotein by a bile acid sequestering resin, cholebine (MCI-196) in type II hyperlipoproteinemia. Atherosclerosis 129: 241–248. 9105567 10.1016/s0021-9150(96)06034-0

[27] Picard F, Gehin M, Annicotte J, Rocchi S, Champy MF, et al (2002) SRC-1 and TIF2 control energy balance between white and brown adipose tissues. Cell 111: 931–941. 12507421 10.1016/s0092-8674(02)01169-8

[28] Mataki C, Magnier BC, Houten SM, Annicotte JS, Argmann C, et al (2007) Compromised intestinal lipid absorption in mice with a liver-specific deficiency of liver receptor homolog 1. Mol Cell Biol 27: 8330–8339. 17908794 10.1128/MCB.00852-07 PMC2169191

[29] Sakakura H, Suzuki M, Kimura N, Takeda H, Nagata S, et al (1993) Simultaneous determination of bile acids in rat bile and serum by high-performance liquid chromatography. J Chromatogr 621: 123–131. 8294533 10.1016/0378-4347(93)80087-k

[30] Folch J, Lees M, Sloane-Stanley GH (1957) A simple method for the isolation and purification of total lipids from animal tissues. J Biol Chem 226: 497–509. 13428781

[31] van Marken Lichtenbelt WD, Vanhommerig JW, Smulders NM, Drossaerts JM, Kemerink GJ, et al (2009) Cold-activated brown adipose tissue in healthy men. N Engl J Med 360: 1500–1508. 19357405 10.1056/NEJMoa0808718

[32] Cypess AM, Lehman S, Williams G, Tal I, Rodman D, et al (2009) Identification and importance of brown adipose tissue in adult humans. N Engl J Med 360: 1509–1517. 19357406 10.1056/NEJMoa0810780 PMC2859951

[33] Virtanen KA, Lidell ME, Orava J, Heglind M, Westergren R, et al (2009) Functional brown adipose tissue in healthy adults. N Engl J Med 360: 1518–1525. 19357407 10.1056/NEJMoa0808949

[34] Suzuki T, Oba K, Igari Y, Watanabe K, Matsumura N, et al (2012) Effects of bile-acid-binding resin (colestimide) on blood glucose and visceral fat in Japanese patients with type 2 diabetes mellitus and hypercholesterolemia: an open-label, randomized, case-control, crossover study. J Diabetes Complications 26: 34–39. 22240263 10.1016/j.jdiacomp.2011.11.008

[35] Garg A, Grundy SM (1994) Cholestyramine therapy for dyslipidemia in non-insulin-dependent diabetes mellitus. A short-term, double-blind, crossover trial. Ann Intern Med 121: 416–422. 8053615 10.7326/0003-4819-121-6-199409150-00004

[36] Bays HE, Goldberg RB, Truitt KE, Jones MR (2008) Colesevelam hydrochloride therapy in patients with type 2 diabetes mellitus treated with metformin: glucose and lipid effects. Arch Intern Med 168: 1975–1983. 18852398 10.1001/archinte.168.18.1975

[37] Fonseca VA, Rosenstock J, Wang AC, Truitt KE, Jones MR (2008) Colesevelam HCl improves glycemic control and reduces LDL cholesterol in patients with inadequately controlled type 2 diabetes on sulfonylurea-based therapy. Diabetes Care 31: 1479–1484. 18458145 10.2337/dc08-0283 PMC2494667

[38] Goldberg RB, Fonseca VA, Truitt KE, Jones MR (2008) Efficacy and safety of colesevelam in patients with type 2 diabetes mellitus and inadequate glycemic control receiving insulin-based therapy. Arch Intern Med 168: 1531–1540. 18663165 10.1001/archinte.168.14.1531

[39] Zieve FJ, Kalin MF, Schwartz SL, Jones MR, Bailey WL (2007) Results of the glucose-lowering effect of WelChol study (GLOWS): a randomized, double-blind, placebo-controlled pilot study evaluating the effect of colesevelam hydrochloride on glycemic control in subjects with type 2 diabetes. Clin Ther 29: 74–83. 17379048 10.1016/j.clinthera.2007.01.003

[40] Kajiyama G, Tazuma S, Yamashita G, Ochi H, Miura H, et al (1996) Effect of MCI-196 on biliary lipids metabolism in patients with hypercholesterolemia. J Clin Ther Med 12: 1349–1359. 10.1016/s0149-2918(98)80057-x 9663363

[41] Garbutt JT, Kenney TJ (1972) Effect of cholestyramine on bile acid metabolism in normal man. J Clin Invest 51: 2781–2789. 5080408 10.1172/JCI107100 PMC292426

[42] Tomlinson E, Fu L, John L, Hultgren B, Huang X, et al (2002) Transgenic mice expressing human fibroblast growth factor-19 display increased metabolic rate and decreased adiposity. Endocrinology 143: 1741–1747. 11956156 10.1210/endo.143.5.8850

[43] Katsuma S, Hirasawa A, Tsujimoto G (2005) Bile acids promote glucagon-like peptide-1 secretion through TGR5 in a murine enteroendocrine cell line STC-1. Biochem Biophys Res Commun 329: 386–390. 15721318 10.1016/j.bbrc.2005.01.139

[44] Kogire M, Gomez G, Uchida T, Ishizuka J, Greeley GH Jr, et al (1992) Chronic effect of oral cholestyramine, a bile salt sequestrant, and exogenous cholecystokinin on insulin release in rats. Pancreas 7: 15–20. 1557341 10.1097/00006676-199201000-00003

[45] Koop I, Fellgiebel A, Koop H, Schafmayer A, Arnold R (1988) Effect of cholestyramine on plasma cholecystokinin and pancreatic polypeptide levels, and exocrine pancreatic secretion. Eur J Clin Invest 18: 517–523. 3147905 10.1111/j.1365-2362.1988.tb01050.x

[46] Shido O, Yoneda Y, Nagasaka T (1989) Changes in brown adipose tissue metabolism following intraventricular vasoactive intestinal peptide and other gastrointestinal peptides in rats. Jpn J Physiol 39: 359–369. 2796018 10.2170/jjphysiol.39.359

[47] Yoshimatsu H, Egawa M, Bray GA (1992) Effects of cholecystokinin on sympathetic activity to interscapular brown adipose tissue. Brain Res 597: 298–303. 1473000 10.1016/0006-8993(92)91486-x

[48] Brufau G, Bahr MJ, Staels B, Claudel T, Ockenga J, et al (2010) Plasma bile acids are not associated with energy metabolism in humans. Nutr Metab (Lond) 7: 73. 20815878 10.1186/1743-7075-7-73 PMC2942888

[49] Brufau G, Stellaard F, Prado K, Bloks VW, Jonkers E, et al (2010) Improved glycemic control with colesevelam treatment in patients with type 2 diabetes is not directly associated with changes in bile acid metabolism. Hepatology 52: 1455–1464. 20725912 10.1002/hep.23831

