# Supplementary material for: Bile Acid Binding Resin Improves Metabolic Control through the Induction of Energy Expenditure
Source: PLoS One. 2012 Aug 29;7(8):e38286. doi: 10.1371/journal.pone.0038286 (PMC3430641; doi:10.1371/journal.pone.0038286)
Supplement: Figure S1 — BABR improves metabolic control in KK- Ay mice. (A) Body weight (BW) and food intake change of KK-Ay mice. Ch denotes chow, COL denotes chow+colestimide and CHO denotes chow+cholestyramine. (B) A comparison of the weight of liver, epididymal WAT (epWAT) and BAT fat pads after the different interventions. (C) Serum levels of triglycerides (TG), free fatty acids (FFA), total cholesterol (T-C) in KK-Ay mice on the indicated treatments. (D) Serum levels of glucose and insulin in KK-Ay mice on the indicated treatments. The HOMA-IR is calculated as described in the materials and methods. (E) Glucose levels during an OGTT and IPITT, and area under the curve (AUC) and integrated areas under the curve (iAUC) in KK-Ay mice in the different treatment groups. The OGTT were performed after an overnight fast after 2 weeks of administration. Glucose was administered by gavage at a dose of 1 g/kg. The IPITT were performed after 4 hours fast after 3 weeks of administration. Insulin was injected at a dose of 0.75 U/kg. Data are expressed as the mean +/− SEM (n = 5–6). # (P<0.05) or ## (P<0.01) versus Ch. Further description about the materials and methods of the experiments is included in “Materials and Methods S1”. (DOC) [file pone.0038286.s001.doc]

**Supplemental Materials and Methods**

***Materials*.** Cholestyramine was obtained from Sigma (St. Quentin Fallavier, France). Colestimide was a generous gift of Mitsubishi Pharmaceuticals.

***Animal studies*.** Male KK-*A^y^* mice, 6-7 weeks of age, were obtained from CLEA Japan Inc. (Tokyo, Japan). All mice were maintained in a temperature-controlled (23°C) facility with a 12 hours light/dark cycle and were given free access to food and water. The control diet contained 16.8% protein, 73.5% carbohydrate and 4.8% fat. For treatment with BABR, colestimide (2% w/w) or cholestyramine (2.5% w/w) were mixed with diets. The mice were fasted 4 hours before harvesting blood for subsequent blood measurements.

***Clinical biochemistry and evaluation of glucose and lipid homeostasis*.** An OGTT was performed in animals that were fasted overnight. Glucose was administered by gavage at a dose of 1 g/kg. An IPITT was done in 4h fasted animals. Insulin was injected at a dose of 0.75 U/kg. Glucose quantification was done with the Maxi Kit Glucometer 4 (Bayer Diagnostic, Puteaux, France) or Glucose RTU (bioMérieux Inc., Marcy l'Etoile, France). Plasma insulin concentrations were measured using ELISA for mouse (Cristal Chem Inc., Downers Grove, IL). Free fatty acids, triglycerides, and total cholesterol were determined by enzymatic assays (Roche, Mannheim, Germany).

***Statistical analysis*.** Values were reported as mean +/- standard error (SEM). Statistical differences were determined using ANOVA (Statsview software, Abacus concepts, Inc., Berkeley, CA). Statistical significance is displayed as ^#^ (*P* < 0.05) or ^##^ (*P* < 0.01) versus Ch.

**Supplemental Results**

***BABR improves the metabolic profile in genetic mouse models of obesity and diabetes.***

We evaluated the metabolic effects of BABR by administrating either colestimide (COL) (2% w/w) or cholestyramine (CHO) (2.5% w/w) during 22 days to KK-*A^y^* mice, a well-established mouse model of the metabolic syndrome. Animals receiving either colestimide or cholestyramine gained less weight than controls, whereas food intake was not affected by both BABR (Fig. S1A). At necropsy, liver and epididymal white adipose tissue (epWAT) weight was significantly reduced (Fig. S1B). The intrascapular BAT appeared browner, indicative of decreased fat accumulation (not shown). Serum triglycerides, free fatty acids and total cholesterol levels were all significantly reduced by colestimide (Fig. S1C). Cholestyramine decreased serum triglycerides and free fatty acids. Furthermore, fasting serum glucose and insulin levels were also significantly decreased by both BABRs (Fig. S1D). Insulin resistance, as assessed using the homeostasis model assessment for insulin resistance (HOMA-IR), was markedly improved (Fig. S1D). During oral glucose tolerance test (OGTT), KK-*A^y^* mice on BABRs had lower glucose levels at all time points after the glucose administration. In the IPITT, the BABR reduced blood glucose level, but the improvement rate described in iAUC was not affected by the BABRs administration (Fig. S1E).
