# Supplementary material for: Bile Acid Binding Resin Improves Metabolic Control through the Induction of Energy Expenditure
Source: PLoS One. 2012 Aug 29;7(8):e38286. doi: 10.1371/journal.pone.0038286 (PMC3430641; doi:10.1371/journal.pone.0038286)
Supplement: Text S1 — (DOCX) [file pone.0038286.s002.docx]

**Text S1**

***BABR improves the metabolic profile in genetic mouse models of obesity and diabetes.***

We evaluated the metabolic effects of BABR by administrating either colestimide (COL) (2% w/w) or cholestyramine (CHO) (2.5% w/w) during 22 days to KK-*A^y^* mice, a well-established mouse model of the metabolic syndrome. Animals receiving either colestimide or cholestyramine gained less weight than controls, whereas food intake was not affected by both BABR (Fig. S1A). At necropsy, liver and epididymal white adipose tissue (epWAT) weight was significantly reduced (Fig. S1B). The intrascapular BAT appeared browner, indicative of decreased fat accumulation (not shown). Serum triglycerides, free fatty acids and total cholesterol levels were all significantly reduced by colestimide (Fig. S1C). Cholestyramine decreased serum triglycerides and free fatty acids. Furthermore, fasting serum glucose and insulin levels were also significantly decreased by both BABRs (Fig. S1D). Insulin resistance, as assessed using the homeostasis model assessment for insulin resistance (HOMA-IR), was markedly improved (Fig. S1D). During oral glucose tolerance test (OGTT), KK-*A^y^* mice on BABRs had lower glucose levels at all time points after the glucose administration. In the IPITT, the BABR reduced blood glucose level, but the improvement rate described in iAUC was not affected by the BABRs administration (Fig. S1E).
