# Supplementary material for: Bile Acid Binding Resin Improves Metabolic Control through the Induction of Energy Expenditure
Source: PLoS One. 2012 Aug 29;7(8):e38286. doi: 10.1371/journal.pone.0038286 (PMC3430641; doi:10.1371/journal.pone.0038286)
Supplement: Materials and Methods S1 — (DOC) [file pone.0038286.s003.doc]

**Materials and Methods S1**

***Materials*.** Cholestyramine was obtained from Sigma (St. Quentin Fallavier, France). Colestimide was a generous gift of Mitsubishi Pharmaceuticals.

***Animal studies*.** Male KK-*A^y^* mice, 6-7 weeks of age, were obtained from CLEA Japan Inc. (Tokyo, Japan). All mice were maintained in a temperature-controlled (23°C) facility with a 12 hours light/dark cycle and were given free access to food and water. The control diet contained 16.8% protein, 73.5% carbohydrate and 4.8% fat. For treatment with BABR, colestimide (2% w/w) or cholestyramine (2.5% w/w) were mixed with diets. The mice were fasted 4 hours before harvesting blood for subsequent blood measurements.

***Clinical biochemistry and evaluation of glucose and lipid homeostasis*.** An OGTT was performed in animals that were fasted overnight. Glucose was administered by gavage at a dose of 1 g/kg. An IPITT was done in 4h fasted animals. Insulin was injected at a dose of 0.75 U/kg. Glucose quantification was done with the Maxi Kit Glucometer 4 (Bayer Diagnostic, Puteaux, France) or Glucose RTU (bioMérieux Inc., Marcy l'Etoile, France). Plasma insulin concentrations were measured using ELISA for mouse (Cristal Chem Inc., Downers Grove, IL). Free fatty acids, triglycerides, and total cholesterol were determined by enzymatic assays (Roche, Mannheim, Germany).

***Statistical analysis*.** Values were reported as mean +/- standard error (SEM). Statistical differences were determined using ANOVA (Statsview software, Abacus concepts, Inc., Berkeley, CA). Statistical significance is displayed as ^#^ (*P* < 0.05) or ^##^ (*P* < 0.01) versus Ch.
